# Can Glatiramer Acetate Prevent Cognitive Impairment by Modulating Oxidative Stress in Patients with Multiple Sclerosis?

**DOI:** 10.3390/ph17040459

**Published:** 2024-04-03

**Authors:** Anna Gil-Sánchez, Hugo Gonzalo, Marc Canudes, Lara Nogueras, Cristina González-Mingot, Petya Valcheva, Pascual Torres, Jose Carlos Serrano, Silvia Peralta, Maria José Solana, Luis Brieva

**Affiliations:** 1 Institut de Recerca Biomèdica de Lleida (IRBLleida), 25198 Lleida, Spain; hgonzalob@saludcastillayleon.es (H.G.); marc.ccss@icloud.com (M.C.); lara.noguerasp@gmail.com (L.N.); cgonzalezm.lleida.ics@gencat.cat (C.G.-M.); pvladeva@irblleida.cat (P.V.); speralta.lleida.ics@gencat.cat (S.P.); solanamoga@gmail.com (M.J.S.); 2 Hospital Universitario Arnau de Vilanova de Lleida (HUAVLleida), 25198 Lleida, Spain; 3 Neuroimmunology Group, Department of Medicine, University of Lleida, 25198 Lleida, Spain; ptorres@irblleida.cat; 4 NUTREN-Nutrigenomics, Department of Experimental Medicine, University of Lleida, 25198 Lleida, Spain; josecarlos.serrano@udl.cat

**Keywords:** cognitive impairment, cognitive preservation, oxidative stress, glatiramer acetate, antioxidant capacity

## Abstract

Multiple sclerosis (MS) is an autoimmune disease characterized by demyelination and neuroinflammation, often accompanied by cognitive impairment. This study aims (1) to investigate the potential of glatiramer acetate (GA) as a therapy for preventing cognitive decline in patients with MS (pwMS) by modulating oxidative stress (OS) and (2) to seek out the differences in cognition between pwMS in a cohort exhibiting good clinical evolution and control subjects (CS). An exploratory, prospective, multicentre, cross-sectional case–control study was conducted, involving three groups at a 1:1:1 ratio—41 GA-treated pwMS, 42 untreated pwMS, and 42 CS. The participants performed a neuropsychological battery and underwent venepuncture for blood sampling. The inclusion criteria required an Expanded Disability Status Scale score of ≤3.0 and a minimum of 5 years of MS disease. Concerning cognition, the CS had a better performance than the pwMS (*p* = <0.0001), and between those treated and untreated with GA, no statistically significant differences were found. Regarding oxidation, no statistically significant differences were detected. Upon categorizing the pwMS into cognitively impaired and cognitively preserved groups, the lactate was elevated in the pwMS with cognitive preservation (*p* = 0.038). The pwMS exhibited a worse cognitive performance than the CS. The pwMS treated with GA did not show an improvement in oxidation. Lactate emerged as a potential biomarker for cognitive preservation.

## 1. Introduction

Multiple sclerosis (MS) is a chronic, autoimmune-mediated, demyelinating disease which affects the central nervous system (CNS), causing axonal degeneration and astrogliosis. Its impact extends to various CNS regions, including often underestimated cortical areas that conventional magnetic resonance imaging (MRI) may not fully capture [1,2]. This complexity manifests through a spectrum of signs and symptoms, encompassing not only physical alterations, but also cognitive impairment (CI). Despite being a prevalent manifestation in MS, CI has only recently garnered research attention [2]. In fact, more than half the patients with MS suffer from some degree of CI [3], and the most frequently affected cognitive domains are their information processing speed, attention, memory, and executive functions (EFs) [2], which are potential sources of disability affecting work skills, social interactions, coping strategies, and the quality of life of patients and their families [4]. Furthermore, truly little is known about the cognitive status of patients with MS (pwMS) with a benign course, forgetting this aspect in this form of the disease. One explanation is that cognitive impairment tends to worsen with the evolution of the disease, but it may also be relevant during the initial stages [2]. Another explanation is that the mental state on the Expanded Disability Status Scale (EDSS) or Kurzke scale is undervalued [5]. It should also be noted that CI is a multifactorial phenomenon that is difficult to measure. This is why it has been described that CI is related, inter alia, to (1) grey matter involvement (cortical lesions), (2) white matter lesions (due to demyelination) that give rise to progressive axonopathy, (3) age (neurosenescence), (4) comorbidities, (5) cognitive reserve, (6) the consumption of toxic substances, (7) feeding habits, and (8) Epstein–Barr virus and vitamin D [6,7,8]. Although CI’s explanation is multifactorial, there is one topic that deserves special mention: oxidative stress (OS).

OS influences the degree of cognitive impairment in other neurodegenerative diseases, such as Alzheimer’s and Parkinson’s diseases [9,10]. Hence, OS might be involved in cognition in MS. In general, the pathomechanisms of excitotoxicity are complex and involve glutamate overload, ionic channel dysfunction, calcium overload, mitochondriopathy, proteolytic enzyme production, and the activation of apoptotic pathways [11]. In MS, the OS levels are related to the progression of MS. There are studies that suggest that the loss of myelin from nerve sheaths is possible because the immune system also participates, along with defects in the mitochondria, in the generation of nitrogen and oxygen free radicals. Macrophages and monocytes release oxidative stress mediators into myelin, and lymphocytes attack it directly [12,13,14].

In summary, the brain is the paramount energy consumer in the human body, and any mitochondrial impairment leading to energy insufficiency could significantly impact neurons and their interconnections. Such repercussions are anticipated to be reflected in one’s cognition. Studies conducted in our laboratory have shown an increase in the cerebrospinal fluid levels of oxidative damage markers in pwMS [15]. However, a direct association between OS and CI in pwMS has not yet been demonstrated.

Previous studies that have carried out disease-modifying treatments (DMTs) and investigated their effects on cognition point to a benefit for cognitive performance by reducing the development of new lesions and the progression of cerebral atrophy [16,17]. However, new studies with DMTs have shown stability or a mild improvement in cognitive outcomes in a short-time follow-up (the duration of most studies is ≤2 years) [18]. Nevertheless, the studies do not report a standard cognitive evaluation and the placebo effect, the possible effect of other CNS drugs, and the time needed under DMTs to prevent CI are unclear in most of the observations [17,18].

The amino acid copolymer glatiramer acetate is an approved treatment for MS, with a singular mode of action. The dosage can be chosen between two modalities: 20 mg (one prefilled syringe) administered as a subcutaneous injection once daily, or 40 mg (one prefilled syringe) administered as a subcutaneous injection three times a week separated by at least 48 h. GA affects various levels of the innate and the adaptive immune response, generating a change from the pro-inflammatory to the anti-inflammatory pathway. This immunomodulatory effect has neuroprotective and repair consequences in the central nervous system [19]. Due to these characteristics, we propose GA as a suitable candidate to analyse its possible benefits in the cognition of pwMS. However, GA has not yet demonstrated a clear effect on cognition. In 1997, a 2-year longitudinal study showed improvements in neuropsychological test scores during 2 years of treatment, regardless of whether the patients received GA or placebo [20]. A follow-up of this study was conducted over a period of 10 years. It showed that the mean scores of tests of memory and semantic retrieval did not significantly change for most patients and worsened in 19% of cases. However, it must be considered that in the follow-up, there was no longer a placebo group, so we do not know how the untreated patients would have behaved after 10 years of illness [21]. What is more, as mentioned, in recent years, the observed trend supports the hypothesis that GA could be a preventive drug against cognitive decline. The studies performed with experimental autoimmune encephalomyelitis (EAE) revealed a significant progressive memory decline and that GA, administered at the time of immunization, partially guarded against rapid memory decline and GA-treated mice performed significantly better than EAE-untreated mice [22,23]. In addition, a systematic review has summarized the positive effects in pre-clinical and clinical studies of GA not only in MS, but also in other pathologies such as Alzheimer’s and Parkinson’s disease. Thus, recent studies have found that GA has more therapeutic benefits than previously thought, with emerging evidence that GA immunomodulation induces cerebral BDNF and IGF-1 expression, reduces OS, and has neuroprotective effects in the central nervous system [19]. Moreover, GA reduces OS in peripheral blood adherent mononuclear cells [24], reinforcing the hypothesis of antioxidant effects as a mechanism of action.

The role of OS, potentially regulated by DMTs, in MS evolution, particularly in CI, remains unknown. Based on the above, we propose that treatment with GA could be a modulator of OS and may thus protect against cognitive impairment in pwMS. Furthermore, we believe that cognitive impairment in patients with good clinical evolution is much more prevalent than that in control subjects.

## 2. Results

### 2.1. Sample Description

A summary of the clinical and sociodemographic data is presented in Table 1. No differences were found in the sociodemographic characteristics of the three groups (gender, age, years of education), nor in the years of MS evolution and EDSS (for the pwMS groups).

### 2.2. Differences in Patient’s Cognition 

**Figure 1:**
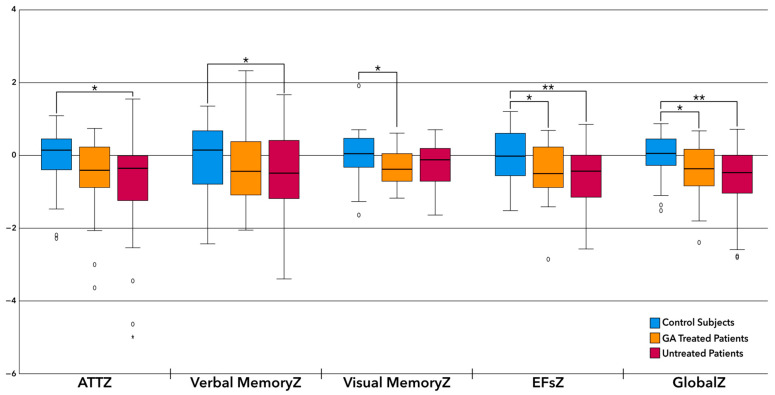
Cognition: Differences among GA-treated pwMS, untreated pwMS, and CS. * *p* < 0.05, ** *p* < 0.05. ATTZ: attention domain Z scores, Verbal MemoryZ: verbal memory domain Z scores, Visual MemoryZ: visual memory domain Z scores, EFsZ: executive functions domain Z scores, GlobalZ: global cognition index Z scores.

Post hoc differences, analysed with the Bonferroni test, were found between the pwMS and CS. Patients obtained a worse performance than the CS. Greater differences were observed when comparing the CS to the untreated pwMS, Figure 1.

### 2.3. Antioxidant Capacity

Table 2 presents a description of the differences between the pwMS and CS regarding the antioxidant capacity using different methods. The levels of lactate and uric acid in plasma are also described. There were no significant differences among the groups.

The differences in the oxidation capacity between patients with MS who were classified as cognitively impaired and cognitively preserved can see in Table 3. We observed higher levels of lactate in the cognitively preserved patients and in the CS regarding cognitive impairment.

### 2.4. Relationship between Cognition, Treatment, and Antioxidant Capacity

**Figure 2:**
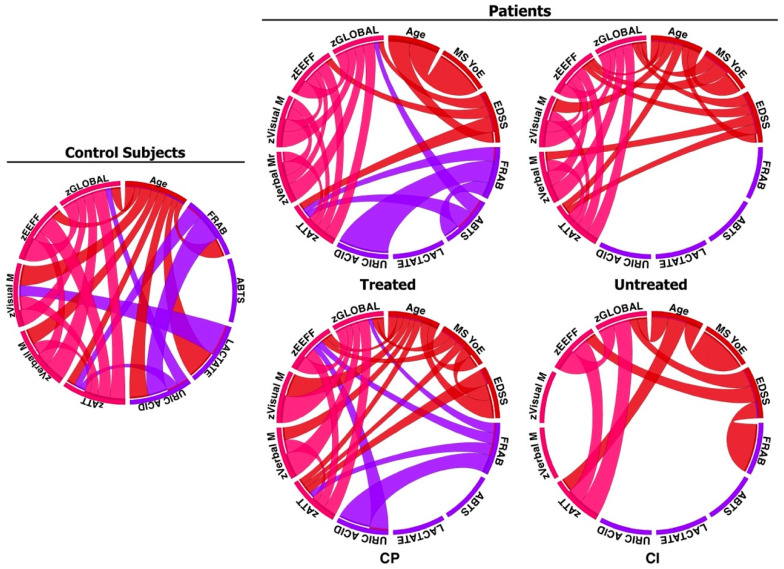
Correlations among the variables were evaluated in each of the groups: The pink lines refer to the significant correlations that arose from the cognition variables. The red lines refer to the clinical and sociodemographic variables. The purple lines refer to the oxidation variables.

Figure 2 presents the analysis of the correlations between the cognitive domains and a higher/lower antioxidant capacity in the CS, GA-treated patients, untreated patients, and the cognitively impaired and cognitively preserved groups. 

In the CS group, there were inverse correlations between attention and FRAP, as well as attention and uric acid. Thus, a lower antioxidant capacity was associated with more attention. Moreover, there was a positive correlation between visual memory and lactate: the higher the visual memory score, the higher the lactate score. There was also an inverse correlation with global cognition and uric acid: the better the cognition, the lower the uric acid index. For untreated MS patients, inverse correlations were observed between FRAP and attention. There was a positive correlation between ABTS and attention and global cognition. There were no significant correlations in the patients between cognitive impairment and oxidation. The correlations between cognitively preserved patients and oxidation were inverse, as in the healthy controls: better attention, less FRAP; better performance in EFs, less FRAP; and the better the global cognition, the less FRAP. The same occurred with uric acid: less uric acid was associated with better overall cognition.

## 3. Discussion

There are two main findings in this study: One is the differences observed in cognition between the pwMS and CS even with good clinical MS evolution. The second is the role of lactate as a possible biomarker of cognitive preservation. 

Regarding the initial finding, it could be elucidated by considering that while many of the patients included in the study exhibited disease characteristics consistent with the definition of benign multiple sclerosis (MS), characterized by minimal physical disability over an extended time period, they nonetheless experienced other less overt symptoms, such as fatigue, depression, and anxiety. This raises questions about the validity of defining benign MS solely based on the physical disability criteria [25,26]. These symptoms could be modulating or worsening cognition; however, cognitive impairment may also be present from the initial stages of the disease but detected only with more sensitive tools [27]. The cognitive state has been related to the neurodegeneration observed in the thickness of the retinal nerve fibre layer using optical coherence tomography [28] in the increased third ventricle width [29], or in the decrease in the thalamic volume using magnetic resonance imaging [30]. All these parameters have been detected at the onset of MS. This means that in many cases, if the symptoms of the disease do not manifest, there are subclinical parameters indicating that the disease is not benign, cognition being one of them. Therefore, cognitive impairment, although sometimes not manifested in the patient, can be measured and verified at any time during the evolution of the disease. Hence, a neuropsychological examination helps make a correct assessment of the actual disability and guides towards the correct prognosis of the evolution of the disease. 

This is the first time a study has been carried out with the aim to evaluate the effect of GA treatment on cognitive impairment in a cohort of patients who are homogeneous in their clinical characteristics. In summary, the obtained results show differences between the pwMS and CS, but no statistical differences between the treated and untreated pwMS in relation to cognition or OS.

When we analysed the differences between the three study groups, we saw that the treated patients were more similar to the CS, although the levels of performance between the two groups of patients (treated and not treated) did not show any statistically significant differences. This was an interesting result, taking into account that pwMS are normally not treated when their disease has a very good prognosis and benign course. This leads us to think that GA could have a positive impact on cognition even when the illness is associated with little physical disability. We can elucidate the positive impact on cognition through several factors contributing to why some patients with MS do not undergo disease-modifying treatment. These reasons typically include: (1) the rejection of treatment, (2) discontinuation due to ineffectiveness, often observed in advanced stages of the disease where the risk–benefit ratio of immunosuppressive treatments is unfavorable, and (3) opting not to initiate treatment due to favorable prognostic factors. Although the latter reason has diminished in significance over the years, with fewer patients now forgoing MS treatment, there are still cases where this decision has persisted from the outset due to the absence of inflammatory activity or disability progression over time. With the advent of improved therapeutic options and a paradigm shift favoring early and continuous treatment, a subset of MS patients has maintained a decision not to undergo treatment due to their inherently benign disease course. This subset represents the profile of untreated MS patients included in our study. This benign MS group is juxtaposed with another group wherein treatment with GA was initiated from the outset due to the presence of worse prognostic factors. Over time, patients treated with GA, despite initially presenting a worse prognosis, have managed to achieve a similarly benign disease course as untreated patients. This outcome is regarded as a success of GA treatment, considering that the initial profile of these patients indicated a poorer prognosis. Consequently, the group of patients treated with GA has evolved in a manner mirroring that of benign MS, reflecting the best possible outcome.

However, the positive benefits of GA are not through the modulation of OS, but through its anti-inflammatory and neuroprotective effect. In fact, GA induces a broad immunomodulatory effect. This includes competition for MHC binding; antagonism at specific T-cell receptors; biases of dendritic cells, monocytes, and B-cells toward anti-inflammatory responses; the induction of Th2/3 and T-regulatory cells; and the downregulation of Th1 and Th-17 cells. GA-specific immune cells penetrate the CNS, secrete in situ anti-inflammatory cytokines, and induce the bystander immunomodulation of the resident cells. GA treatment generates neuroprotective repair consequences, such as neurotrophic factors secretion including brain-derived neurotrophic factor, neurotrophin-3, neurotrophin-4, insulin-like growth factor 1, and insulin-like growth factor 2, as well as reduced myelin and neuroaxonal damages, remyelination, and neurogenesis. These findings imply that GA immunomodulation may affect the neurodegenerative and cognition aspects of MS as well as of other CNS pathologies including Parkinson’s disease, Alzheimer’s disease, and lateral sclerosis [19,31]. 

Regarding OS, no statistically significant differences were found among the parameters explored when we compared the CS, treated, and untreated pwMS. Although the treated group had parameters similar to the control group, once more, there was no statistically significant difference with respect to the untreated group. This could be because, in these cases with scant disability, antioxidant mechanisms are not still necessary to compensate for OS. However, it could be that in more advanced stages, we observed an imbalance. In a recent study by our team [32], an increase in the levels of superoxide production in PBMCs (peripheral blood mononuclear cells) was found in pwMS, even with an increase in the lactate plasma levels, but these findings have not been replicated in the current study, probably because the characteristics of the samples are different, with a cohort of older and patients with a higher level of disability in the past study. To gain more insight into these phenomena, it might be possible to replicate a second observation study with the same cohort after 5–10 years of the first study. In addition, in future studies, we want to extend the evaluation of oxidation by adding enzymatic activity, such as NOX2, glutathione peroxidase, superoxide dismutase, and catalase.

When we analysed the differences between MS patients with a defined cognitive impairment and cognitively preserved patients, we noted that lactate could be considered as a biomarker for cognition: its levels were higher in the pwMS with cognitive preservation, with similar levels to the CS and treated pwMS, and the cognitively impaired pwMS had the lowest lactate values. Lactate is the final product of the glucose metabolism; it comes from anaerobic exercise and it is used as a source of energy. Some authors have described elevated levels of lactate in pwMS that could arise after exercise or from a lack of elimination [33,34]. Regarding lactate in cerebrospinal fluid, an elevation of lactate has been shown in relapsing–remitting MS vs. healthy subjects, and there is an increase in lactate when the rate of the progression of the patient increases, although with an inverse correlation with the duration of the disease [35]. In studies examining lactate indices in peripheral blood, it has been observed that resting lactate levels are elevated in pwMS compared to control subjects. Furthermore, during exercise, these patients exhibited a diminished ability to increase their lactate levels compared to healthy individuals. This suggests a potential alteration in lactate metabolism as an alternative energy source in MS patients. However, it has been noted that these indices show improvement following sustained training, indicating a potential for improvement in the underlying mechanism [34]. 

Even more interesting is that there are studies that have observed lactate indices not only during exercise, but also during cognitive tasks. These new studies have correlated lactate levels with a better cognitive performance [36,37]. The mechanism underlying this phenomenon is that all types of glial cells contribute to the processing of encoded and stored information. As the brain requires a large amount of energy for cognitive tasks, the use of glucose metabolism is not always sufficient for complex cognitive performance, where the role of lactate and its support for neurons through astrocytes becomes relevant [37]. The above-mentioned studies support the idea that lactate is necessary for cognitive tasks requiring a high attentional load, such as object-in-place tasks, but not for less demanding tasks, such as simple novel object recognition. These new findings are in line with our results explaining why the pwMS with cognitive impairment had worse levels than the rest of the groups. Furthermore, except for a small number of cases, all blood extractions were performed after the neuropsychological examination and were not conducted in fasting conditions. However, we must mention that the amount of exercise the patients engaged in daily or weekly was not measured. These factors could change the lactate levels in our patients. In our cohort, it is possible that low levels of lactate in patients with cognitive impairment may indicate that the compensatory energetic mechanisms involving lactate in neurons and support cells are dysfunctional as a consequence of a severe impairment where progression primarily manifests as cognitive decline.

Regarding the correlations between OS and cognition, both the treated patients and CS exhibited inverse correlations between the cognitive domains and antioxidant defences. This again suggests that these groups behave in a comparable way, while the untreated patients did not follow the same trend. This could be because the compensatory oxidation mechanisms are not necessary in these groups with low or no disability. However, our results do not replicate those from similar studies published in the scientific literature. The research has found a lower antioxidant capacity of plasma in patients with Alzheimer’s disease and controls expressing the APOE genotype 4/4 without cognitive damage, but have not found a lower antioxidant capacity when the subjects performed cognitive tasks better [10]. In a past study, we found a decreased antioxidant capacity in the plasma of pwMS, although half of them had a worse clinical course and EDSS [32]. Nevertheless, regarding visual memory and CS, again, the lactate correlates positively with cognition, showing that the better the results of visual memory, the higher the blood lactate levels. The effect of antioxidant defences will probably not be observed until the neurodegenerative damage is very evident or until the difficulty of the task requires the performance of this extra support. 

The correlations between uric acid and general cognition (global Z) were, once again, inverse. Recent studies have demonstrated that uric acid may exert neuroprotective actions in Alzheimer’s and Parkinson’s diseases, with low levels of uric acid representing a risk factor for quicker disease progression and a possible marker of malnutrition. Conversely, high serum uric acid may negatively influence the disease course in vascular dementia [38]. These negative results would be more in line with our findings, but they still have to be further studied. 

In summary, GA can exert a protective effect for cognition, but the mechanism of action would not be the modulation of OS since it seems that the process of the activation of antioxidant defences was not yet underway in our cohort of patients.

One limitation in our study could be that we are correlating quite different methods, such as the biological antioxidant capacity and the estimation of cognitive domains. Nevertheless, we have tried to minimize this phenomenon with the Z score. Other limiting factors in our study are the fact we were not able to measure the effects of nutrition and exercise on the oxidation parameters of the study participants and we were not able to measure whether the difficulty of the cognitive task affected the lactate mechanism. Finally, we did not measure our cohort after MS evolution changes; these will be our next objective for a future study.

## 4. Materials and Methods

### 4.1. Design

**Figure 3:**
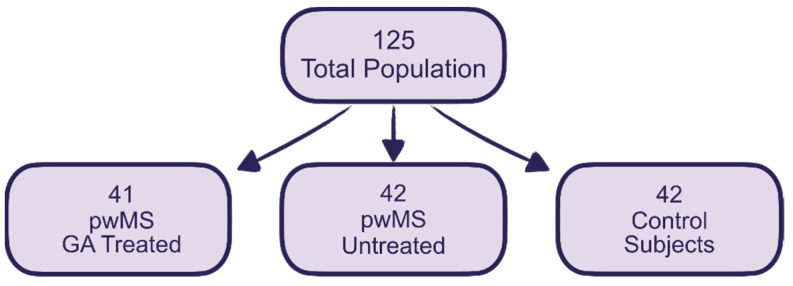
Sample characteristics.

An exploratory, multicentre, cross-sectional case–control study was conducted on a total of 125 volunteers divided into three groups at a ratio of approximately 1:1:1, which included 41 treated pwMS, 42 untreated pwMS, and 42 CS. The rationale for including the latter group was to facilitate a comparison between individuals without multiple sclerosis (MS) and those with MS within our study cohort. The existing literature suggests that individuals with a benign disease course typically exhibit minimal physical disability. However, cognitive impairment is frequently underestimated in this population. Our aim was twofold: firstly, to assess whether patients receiving glatiramer acetate (GA) exhibited any protective effects against cognitive impairment, and secondly, to determine whether their cognitive performance was comparable to that of the control subjects. All three groups were submitted to a neuropsychological study consisting of a G factor test (fluid intelligence), Raven’s progressive matrices [39], and the Brief Repeatable Battery of Neuropsychological Tests (BRB-N) [3,40,41], to which we added the Stroop test, two verbal fluency tests (COWA semantic and phonetic fluency) [42,43], and a trail-making test [44]. All those participating in the trial had a blood sample taken by means of venepuncture. The G factor test was used as a reference for each individual’s general cognitive ability so as to be able to compare this with their performance in the rest of the domains explored, which were attention–concentration, VPI, EFs, and verbal and visual memory. The plasma antioxidant capacity was estimated using the following parameters: a FRAP calculation, ABTS, and uric acid and lactate levels. In order to achieve the desired number of cases and controls, we requested the collaboration of other health centres in Spain. A total of 9 more centres were included. All the data obtained were registered in a single database in compliance with the provisions of Regulation (EU) 2016/679 (GDPR) and the Spanish Organic Law, 3/2018, of 5 December, on the Personal Data Protection and of Digital Rights Guarantee. Recruitment began in March 2015 and ended in June 2021, but had to be suspended from March 2020 to March 2021 due to the COVID-19 pandemic (Figure 3).

We obtained approval from the Arnau de Vilanova University Hospital’s (Lleida, Spain) ethics committee for this study; reference number CEIC-1337. In addition, all patients were informed of all procedures and signed informed consent. The project was conducted in accordance with the basic principles of the protection of the rights and dignity of human beings, as stated in the Declaration of Helsinki and according to the current regulations following Spanish Law 14/2007 on biomedical research for biological samples.

Inclusion criteria:

(1) Control subjects: Subjects not diagnosed with MS or other neurological and/or psychiatric diseases that could compromise their cognitive function. The CS were willing to collaborate in the study without financial benefit.
(2) PwMS treated with GA: patients with a diagnosis of relapsing–remitting MS (RRMS), according to McDonald 2010, whose expanded disability status scale (EDSS) score was ≤3.0 with ≥5 years of MS evolution, and who had been treated with GA for at least the previous year (the two dosages of GA were admitted).
(3) PwMS without treatment: Patients with benign RRMS defined as an EDSS score of ≤3.0 at ≥5 years after the onset of MS. This group did not receive any DMTs, although drug use was accepted to alleviate symptoms of the disease, such as fatigue, pain, or spasms.

Exclusion criteria:

(1) Diagnosis of any other neurodegenerative disease that could involve increased CI or OS.
(2) Diagnosis of a psychiatric illness, drug addiction, or mental disability that would have prevented them from understanding the nature of the study.
(3) Refusal to provide informed consent.

These criteria were chosen to demonstrate that treatment with GA is better at preserving cognition than not taking a DMT, even in patients with features of benign MS, all with a similar EDSS, considering that the EDSS is not a good tool to measure cognition.

### 4.2. Sample Collection

The neuropsychological assessment was carried out in all hospitals involved in the study by two professionals trained and qualified to perform the battery tests. The total battery time was two hours per patient. To determine the presence of CI, it was necessary for the subject to fail in three or more tests in the complete neuropsychological examination. Failure was determined by scores that were one and a half standard deviations below the mean, that is, PC < 5 in the test score. These criteria are recommended in the current MS literature [40,41]. A global Z was created to provide a global index of cognition [45,46].

Peripheral blood samples were collected under non-fasting conditions after completing the neuropsychological performance by nurses from each of the participating hospitals and sent to the MS unit (UEM-Lleida) at HUAV. Cell fractionation was carried out and the plasma samples were conserved at −80 °C until analyses were made by the UEM-Lleida clinical neuroimmunology team. The samples were stored at the Biobank of the Lleida Biomedical Research Institute (IRBLleida). All the samples were processed at the IRBLleida laboratory.

### 4.3. Methods to Measure OS or Antioxidant Capacity (FRAP, ABTS, Uric Acid, and Lactate)

#### 4.3.1. FRAP

The FRAP assay is a typical ET-based (Electron Transfer based) method that measures the reduction of ferric ion (Fe^3+^)–ligand complexes to the intensely blue-colored ferrous ion Fe^2+^ complex by antioxidants in acidic media. Simplified: The FRAP method measures the ferric reduction capacity of the FRAP reagent in the presence of antioxidants. 

The FRAP (ferric-reducing antioxidant power) assay [47,48] was performed by mixing 900 μL of the FRAP reagent (2,4,6-tri(2-pyridyl)-s-triazine, FeCl3, and acetate buffer (300 mM, pH 3.6)) with 90 μL of distilled water and 30 μL of plasma or sodium phosphate buffer for the blank. The measurement was performed in 96-well plates and increasing concentrations between 100 µM and 1000 µM of TROLOX (6-hydroxy-2,5,7,8-tetramethylchroman-2-carboxylic acid, Sigma, ref: 23881-3) were employed to make the standard curve that allowed the antioxidant capacity of each sample to be calculated. TROLOX is an analogue of vitamin E which, therefore, has an antioxidant capacity and is used as a reference antioxidant (standard) in techniques such as this. Then, 3 µL of samples or standard was added to each well, to which 27 µL of PBS and 200 µL of FRAP reagent were added. The plate was mixed and incubated for 30 min at 37 °C and the absorbance was read at 595 nm using a spectrophotometer (Multiskan Ascent 354. Thermo Labsystem, spectralab scientifics, Toronto, ON, Canada).

#### 4.3.2. ABTS

ABTS measures the radical scavenging capacity [10,35]. Originally the ABTS^+^ method was based on the activation of metmyoglobin, acting as peroxidase, with H_2_O_2_ to generate a ferrylmyoglobin radical, which then reacted with ABTS to form an ABTS^+^ radical cation. In terms of assay conditions, different strategies have been implemented for ABTS^+^ generation [48].

To perform the assay, the required volume of oxidized ABTS was diluted in phosphate-buffered saline so that the absorbance was approximately 1 when read at 405 nm. The standard curve was again performed using TROLOX as a standard, and all measurements were performed against a blank composed of 1 µL of PBS to which 200 µL of ABTS reagent was added. A total of 7 measurements were performed for all samples, the first at time 0, and the following 6 at one minute after the previous (minutes 1, 2, 3, 4, 5, and 6). To calculate the antioxidant capacity, the inhibition percentage of each sample was calculated with respect to the TROLOX inhibition percentage, and the area under the curve was calculated [48,49].

#### 4.3.3. Uric Acid

The concentration of uric acid in the plasma (previously frozen at −80 °C) of the study subjects was analysed using a commercial uric acid kit (Spinreact, ref: 1001010). The principle of the method is based on the oxidation of uric acid by uricase. Allantoin and hydrogen peroxide are generated from this reaction which, in the presence of 2,4-dichlorophenol sulfonate and 4-aminophenazone, forms a red compound that is detected by spectrophotometry and generated proportionally to the concentration of uric acid.

The samples were analysed following the manufacturer’s recommendations in 96-well plates, and all measurements were performed in triplicate. A standard curve was prepared with standard concentrations between 1 mg/dL and 6 mg/dL, which allowed the concentrations of the samples to be analysed. The absorbance was analysed at 505 nm [50]. 

#### 4.3.4. Lactate

The determination of the lactate concentration was conducted using a commercial lactate kit (Spinreact, ref: 1001330, Girona, Spain) following the manufacturer’s instructions, but with adapted volumes to carry out the measurement in a plate of 96 wells.

The lactate present in the plasma is oxidized by lactate oxidase to pyruvate and hydrogen peroxide which, in the presence of peroxidase, 4-aminophenazone, and 4-chlorophenol, forms a red product quantifiable by spectrophotometry at 505 nm. A standard curve was made with the concentrations of the standard included between 0 and 10 mg/dL, and the blank (composed only of the working reagent) absorbance was subtracted from all measurements. After adding the samples, standards, and the working reagent, the plate was incubated at room temperature for 10 min and analysed [51].

### 4.4. Statistical Analyses 

For the statistical analysis, we used SPSS 25.0 (IBM: Armonk, NY, USA) and different tests depending on the variables and groups. To analyse the categorical demographic variables, we used the Chi-square test. To analyse the intergroup differences, the normality was checked with the Shapiro–Wilk test and the homoscedasticity with the Levene test. For the variables that met these assumptions, the ANOVA test was applied, and the Bonferroni statistic was used for post hoc analysis. For the rest of the contrasts, the Kruskal–Wallis or Mann–Whitney U test was used, depending on the number of groups to be compared. Finally, we performed a Spearman correlation analysis and used the CIRCOS platform to make the graphs (http://mkweb.bcgsc.ca/tableviewer/visualize/ (accessed on 22 June 2022)). Correlations with a *p*-value of less than 0.05 were selected, and a chromatic classification was assigned according to whether they were demographic (red), cognitive (pink), or oxidative stress (violet) variables.

## 5. Conclusions

The results support the hypothesis that GA could have an influence in preventing cognitive impairment, because the group treated with GA behaved similarly to the CS in terms of cognitive performance, but not in the antioxidant capacity. Furthermore, as the patients who were not treated were those who had better prognoses, with protection from physical disability and perhaps protection against CI, GA could prove its effectiveness by obtaining similar results for untreated patients. However, the differences were not statistically significant, and although the study aimed to consider the choice of the treatment not only for physical disability, but also to protect the patient’s cognition, we can only conclude that GA could be a suitable choice, with limitations. Furthermore, the mechanism underlying this protection would not be the modulation of or decrease in OS.

Additionally, we observed that lactate could be a biological marker for cognitive preservation or better performance, as its levels were higher in the pwMS and CS who had their cognition preserved. 

No other oxidation parameter was observed to be altered or increased in the rest of the patient groups and, therefore, no significant changes were observed in the oxidation of patients with good clinical evolution.

In future studies, it will be necessary to try to elucidate which other variables, such as diet, exercise, general health, or difficulty in the cognitive task, could influence the modulation of oxidation and the consequences for cognitive parameters. It will also be necessary to observe possible changes over time in the same cohort and to extend the evaluation of oxidation by adding enzymatic activity.

## Figures and Tables

**Table 1:** PwMS (treated and untreated) and CS.

	GA-Treated (*n* = 41)	Untreated (*n* = 42)	CS (*n* = 42)	*p*-Value
Sex (*n*, % females)	25 (61%)	33 (79%)	33 (79%)	0.116
Age ($\bar{X}$, SD^5^)	42.05 (9.16)	42.9 (8.56)	38.05 (15.55)	0.125
Years of education ($\bar{X}$, SD)	12.9 (3.1)	12.64 (3.18)	13.83 (3.16)	0.192
MS years of evolution ($\bar{X}$, SD)	10.39 (5.56)	13.02 (7.15)	-	0.072
EDSS (median)	1 (0–3)	1.5 (0–3)	-	0.307
CP vs. CI (*n*/*n*, %CP/%CI)	28/12 (68.3%/31.7%)	26/16 (61.9%/38.1%)	40/2 (95.2%/4.8%)	-
CI: Mild (*n*,%)	3 (7.3%)	6 (14.3%)	2 (4.8%)	-
CI: Moderate (*n*,%)	6 (14.6%)	4 (9.5%)	-	-
CI: Severe (*n*,%)	4 (9.8%)	6 (14.3%)	-	-

$\bar{X}$: mean, CP: cognitively preserved, CI: cognitively impaired, *n*: number of patients, %: percentage, GA: glatiramer acetate, CS: control subjects.

**Table 2:** OS: differences among GA-treated pwMS, untreated pwMS, and CS.

	CS (*n* = 42)	pwMS			
		Total (*n* = 83)	Treated (*n* = 41)	Untreated (*n* = 42)	*p*-Value *
FRAP ($\bar{X}$, SD)	275.86 (138.80)	259.23 (138.15)	288.77 (139.21)	230.39 (132.44)	0.453
ABTS ($\bar{X}$, SD)	551.21 (130.79)	516.77 (155.60)	486.53 (151.74)	546.28 (155.40)	0.500
Lactate ($\bar{X}$, SD)	20.17 (10.98)	17.25 (8.25)	18.27 (8.61)	16.24 (7.86)	0.210
Uric acid ($\bar{X}$, SD)	3.72 (1.81)	3.28 (1.51)	3.57 (1.74)	3.01 (1.21)	0.267

Mann–Whitney U Test. * *p*-values reflect the differences among the pwMS total, treated and untreated pwMS, and CS.

**Table 3:** OS and cognitive impairment: differences between patients classified as cognitively impaired and cognitively preserved.

	CS (*n* = 42)	pwMS CP (*n* = 54)	pwMS CI (*n* = 29)	*p*-Value
FRAP ($\bar{X}$, SD)	275.86 (138.80)	236.59 (110.7)	301.39 (172.83)	0.136
ABTS ($\bar{X}$, SD)	551.21 (130.79)	528.35 (136.5)	495.2 (186.79)	0.724
Lactate ($\bar{X}$, SD)	20.17 (10.98)	18.52 (8.28)	14.87 (7.78)	0.038 *
Uric Acid ($\bar{X}$, SD)	3.72 (1.81)	3.34 (1.66)	3.19 (1.22)	0.879

Mann–Whitney U Test; *p*-value between pwMS CP (patients with multiple sclerosis cognitively preserved) and pwMS CI (patients with multiple sclerosis cognitively impaired). * Statistically significant.

## Data Availability

Data is contained within the article.

## References

[1] Calabrese M., Filippi M., Gallo P. (2010). Cortical lesions in multiple sclerosis. Nat. Rev. Neurol..

[2] Amato M.P., Ponziani G., Siracusa G., Sorbi S. (2001). Cognitive Dysfunction in early-onset Multiple Sclerosis. A Reappraisal after 10 years. Arch. Neurol..

[3] Rao S.M., Leo G.J., Bernardin L., Unverzagt F. (1991). Cognitive dysfunction in multiple sclerosis. I. Frequency, patterns and predicition. Neurology.

[4] Piacentini C., Argento O., Nocentini U. (2023). Cognitive impairment in multiple sclerosis: “classic” knowledge and recent acquisitions. Arq. Neuropsiquiatr..

[5] Kurtzke J.F. (1983). Rating neurologic impairment in multiple sclerosis: An expanded disability status scale (EDSS). Neurology.

[6] Ward M., Goldman M.D. (2022). Epidemiology and Pathophysiology of Multiple Sclerosis. Continuum.

[7] Geurts J.J., Calabrese M., Fisher E., Rudick R.A. (2012). Measurement and clinical effect of grey matter pathology in multiple sclerosis. Lancet Neurol..

[8] Lisak M., Špiljak B., Pašić H., Trkanjec Z. (2021). Cognitive Aspects in Multiple Sclerosis. Psychiatr. Danub..

[9] Zafrilla P., Mulero J., Xandri J.M., Santo E., Caravaca G., Morillas J.M. (2006). Oxidative stress in Alzheimer patients in different stages of the disease. Curr. Med. Chem..

[10] Pulido R., Jiménez-Escrig A., Orensanz L., Saura-Calixto F., Jiménez-Escrig A. (2005). Study of plasma antioxidant status in Alzheimer’s disease. Eur. J. Neurol..

[11] Gonsette R.E. (2008). Neurodegeneration in multiple sclerosis: The role of oxidative stress and excitotoxicity. J. Neurol. Sci..

[12] Flores-Alvarado L.J., Gabriel-Ortiz G., Pacheco-Mois F.P., Bitzer-Quintero K. (2015). Mecanismos patogénicos en el desarrollo de la esclerosis múltiple: Ambiente, genes, sistema inmune y estrés oxidativo [Pathogenic mechanisms of neuronal damage in multiple sclerosis]. Investig. Clin..

[13] Ohl K., Tenbrock K., Kipp M. (2016). Oxidative stress in multiple sclerosis: Central and peripheral mode of action. Exp. Neurol..

[14] Rathore K.L., Kerr B.J., Redensek A., López-Vales R., Jeong S.Y., Ponka P., David S. (2008). Ceruloplasmin protects injured spinal cord from iron-mediated oxidative damage. J. Neurosci..

[15] Gonzalo H., Brieva L., Tatzber F., Jové M., Cacabelos D., Cassanyé A., Lanau-Angulo L., Boada J., Serrano J.C.E., González C. (2012). Lipidome Analysis in Multiple Sclerosis Reveals Protein Lipoxidative Damage as A Potential Pathogenic Mechanism. J. Neurochem..

[16] Patti F., Leone C., D’Amico E. (2010). Treatment options of cognitive impairment in multiple sclerosis. Neurol. Sci..

[17] Landmeyer N.C., Bürkner P.C., Wiendl H., Ruck T., Hartung H.P., Holling H., Meuth S.G., Johnen A. (2020). Disease-modifying treatments and cognition in relapsing-remitting multiple sclerosis: A meta-analysis. Neurology.

[18] Carlomagno V., Mirabella M., Lucchini M. (2023). Current Status of Oral Disease-Modifying Treatment Effects on Cognitive Outcomes in Multiple Sclerosis: A Scoping Review. Bioengineering.

[19] Kasindi A., Fuchs D.T., Koronyo Y., Rentsendorj A., Black K.L., Koronyo-Hamaoui M. (2022). Glatiramer Acetate Immunomodulation: Evidence of Neuroprotection and Cognitive Preservation. Cells.

[20] Weinstein A., Schwid S.R., Schiffer R.B., McDermott M.P., Giang D.W., Goodman A.D. (1999). Neuropsychologic status in multiple sclerosis after treatment with glatiramer. Arch Neurol..

[21] Schwid S.R., Goodman A.D., Weinstein A., McDermott M.P., Johnson K.P., Copaxone Study Group (2007). Cognitive function in relapsing multiple sclerosis: Vminimal changes in a 10-year clinical trial. J. Neurol. Sci..

[22] LoPresti P. (2015). Glatiramer acetate guards against rapid memory decline during relapsing-remitting experimental autoimmune encephalomyelitis. Neurochem. Res..

[23] Aharoni R., Schottlender N., Bar-Lev D.D., Eilam R., Sela M., Tsoory M., Arnon R. (2019). Cognitive impairment in an animal model of multiple sclerosis and its amelioration by glatiramer acetate. Sci. Rep..

[24] Iarlori C., Gambi D., Lugaresi A., Patruno A., Felaco M., Salvatore M., Speranza L., Reale M. (2008). Reduction of free radicals in multiple sclerosis: Effect of glatiramer acetate (Copaxone). Mult. Scler..

[25] Ton A.M.M., Vasconcelos C.C.F., Alvarenga R.M.P. (2017). Benign multiple sclerosis: Aspects of cognition and neuroimaging. Arq. Neuropsiquiatr..

[26] Tallantyre E.C., Major P.C., Atherton M.J., Davies W.A., Joseph F., Tomassini V., Pickersgill T.P., Harding K.E., Willis M.D., Winter M. (2019). How common is truly benign MS in a UK population?. J. NeurolNeurosurg. Psychiatry.

[27] Meca-Lallana V., Gascón-Giménez F., Ginestal-López R.C., Higueras Y., Téllez-Lara N., Carreres-Polo J., Eichau-Madueño S., Romero-Imbroda J., Vidal-Jordana Á., Pérez-Miralles F. (2021). Cognitive impairment in multiple sclerosis: Diagnosis and monitoring. Neurol. Sci..

[28] Esmael A., Elsherif M., Abdelsalam M., Sabry D., Mamdouh M., Belal T. (2020). Retinal thickness as a potential biomarker of neurodegeneration and a predictor of early cognitive impairment in patients with multiple sclerosis. Neurol. Res..

[29] Pflugshaupt T., Geisseler O., Nyffeler T., Linnebank M. (2016). Cognitive Impairment in Multiple Sclerosis: Clinical Manifestation, Neuroimaging Correlates, and Treatment. Semin. Neurol..

[30] Jakimovski D., Bergsland N., Dwyer M.G., Hagemeier J., Ramasamy D.P., Szigeti K., Guttuso T., Lichter D., Hojnacki D., Weinstock-Guttman B. (2020). Long-standing multiple sclerosis neurodegeneration: Volumetric magnetic resonance imaging comparison to Parkinson’s disease, mild cognitive impairment, Alzheimer’s disease, and elderly healthy controls. Neurobiol. Aging.

[31] Prod’homme T., Zamvil S.S. (2019). The Evolving Mechanisms of Action of Glatiramer Acetate. Cold Spring Harb. Perspect. Med..

[32] Gonzalo H., Nogueras L., Gil-Sánchez A., Hervás J.V., Valcheva P., González-Mingot C., Martin-Gari M., Canudes M., Peralta S., Solana M.J. (2019). Impairment of Mitochondrial Redox Status in Peripheral Lymphocytes of Multiple Sclerosis Patients. Front. Neurosci..

[33] Keytsman C., Hansen D., Wens I., Eijnde B.O. (2019). Exercise-induced lactate responses in Multiple Sclerosis: A retrospective analysis. NeuroRehabilitation.

[34] Cerexhe L., Easton C., Macdonald E., Renfrew L., Sculthorpe N. (2022). Blood lactate concentrations during rest and exercise in people with Multiple Sclerosis: A systematic review and meta-analysis. Mult. Scler. Relat. Disord..

[35] Albanese M., Zagaglia S., Landi D., Boffa L., Nicoletti C.G., Marciani M.G., Mandolesi G., Marfia G.A., Buttari F., Mori F. (2016). Cerebrospinal fluid lactate is associated with multiple sclerosis disease progression. J. Neuroinflamm..

[36] Alberini C.M., Cruz E., Descalzi G., Bessières B., Gao V. (2018). Astrocyte glycogen and lactate: New insights into learning and memory mechanisms. Glia.

[37] Dembitskaya Y., Piette C., Perez S., Berry H., Magistretti P.J., Venance L. (2022). Lactate supply overtakes glucose when neural computational and cognitive loads scale up. Proc. Natl. Acad. Sci. USA.

[38] Tana C., Ticinesi A., Prati B., Nouvenne A., Meschi T. (2018). Uric Acid and Cognitive Function in Older Individuals. Nutrients.

[39] Raven J. (1981). Manual for Raven’s Progressive Matrices and Vocabulary Scales.

[40] Amato M.P., Morra V.B., Falautano M., Ghezzi A., Goretti B., Patti F., Riccardi A., Mattioli F. (2018). Cognitive assessment in multiple sclerosis-an Italian consensus. Neurol. Sci..

[41] Sepulcre J., Vanotti S., Hernández R., Sandoval G., Cáceres F., Garcea O., Villoslada P. (2006). Cognitive impairment in patients with multiple sclerosis using the Brief Repeatable Battery-Neuropsychology test. Mult. Scler..

[42] Velázquez-Cardoso J., Marosi-Holczberger E., Rodríguez-Agudelo Y., Yañez-Tellez G., Chávez-Oliveros M. (2014). Recall strategies for the verbal fluency test in patients with multiple sclerosis. Neurologia.

[43] Connick P., Kolappan M., Bak T.H., Chandran S. (2012). Verbal fluency as a rapid screening test for cognitive impairment in progressive multiple sclerosis. J. Neurol. Neurosurg. Psychiatry.

[44] Reitan R.M. (2016). Validity of the Trail Making Test as an Indicator of Organic Brain Damage. Percept. Mot. Ski..

[45] Kalb R., Beier M., Benedict R.H., Charvet L., Costello K., Feinstein A., Gingold J., Goverover Y., Halper J., Harris C. (2018). Recommendations for cognitive screening and management in multiple sclerosis care. Mult. Scler..

[46] De Meo E., Portaccio E., Giorgio A., Ruano L., Goretti B., Niccolai C., Patti F., Chisari C.G., Gallo P., Grossi P. (2021). Identifying the Distinct Cognitive Phenotypes in Multiple Sclerosis. JAMA Neurol..

[47] Benzie I.F., Strain J.J. (1999). Ferric reducing/antioxidant power assay: Direct measure of total antioxidant activity of biological fluids and modified version for simultaneous measurement of total antioxidant power and ascorbic acid concentration. Methods Enzymol..

[48] Gulcin İ. (2020). Antioxidants and antioxidant methods: An updated overview. Arch. Toxicol..

[49] Re R., Pellegrini N., Proteggente A., Pannala A., Yang M., Rice-Evans C. (1999). Antioxidant activity applying an improved ABTS radical cation decolorization assay. Free Radic. Biol. Med..

[50] Sautin Y.Y., Johnson R.J. (2008). Uric acid: The oxidant-antioxidant paradox. Nucleosides Nucleotides Nucleic Acids.

[51] Groussard C., Morel I., Chevanne M., Monnier M., Cillard J., Delamarche A. (2000). Free radical scavenging and antioxidant effects of lactate ion: An in vitro study. J. Appl. Physiol..

